# Surgical outcomes of acute acquired comitant esotropia of adulthood

**DOI:** 10.1186/s12886-020-01793-3

**Published:** 2021-01-18

**Authors:** Dae Hyun Kim, Ha Jeong Noh

**Affiliations:** Department of Ophthalmology, Chosun University Hospital, 365 Pilmundaero, Dong-gu, 61453 Gwang-ju, South Korea

**Keywords:** Acute acquired comitant esotropia, Motor outcome, Sensory outcome

## Abstract

**Background:**

Acute acquired comitant esotropia (AACE) is a type of strabismus characterized by a sudden onset of large angle esotropia with diplopia, which often occurs in children after infancy, teenagers, and young adolescents. However, studies on the surgical outcomes of only adults are rare. The purpose of this article is to analyze the surgical outcomes for adult patients diagnosed with AACE.

**Methods:**

Medical records of 24 patients who had undergone surgery for AACE were retrospectively analyzed. The main outcome measures were the final motor and sensory success rate after surgery and factors affecting motor and sensory outcomes. Motor success was considered alignment within 8 prism diopter (PD) at both near and distance and sensory success was stereoacuity ≥ 60 sec/arc.

**Results:**

The preoperative mean esodeviation angles were 33.1 ± 10.4 PD at distance and 33.3 ± 11.2 PD at near. The mean period of postoperative follow up was 7.5 ± 4.5 months (range 1–8 months). The postoperative mean esodeviation angles at final follow-up time were 3.4 ± 6.1 PD at distance and 3.8 ± 6.7 PD at near. The surgical motor success rate at final follow-up was 79.2% (19/24). The sensory success rate at final follow-up was 50.0% (12/24). The factor affecting the motor outcome was the type of surgery (*p* < 0.05). The factor affecting sensory outcome was postoperative follow-up time (*p* < 0.05).

**Conclusions:**

Surgery type appears to affect surgical motor outcomes in adults with AACE. Although the sensory outcome was favorable, it seems that regaining bifoveal fixation takes time.

## Background

Acute acquired comitant esotropia (AACE) is a type of strabismus characterized by a sudden onset of large angle esotropia with diplopia, which often occurs in children after infancy, teenagers, and young adolescents [1]. The intracranial disease including brain tumor had been reported as the cause of some AACE cases; however, the cause is unclear in most cases [2–4]. A recent study reported that the prevalence of AACE is growing as the near work increased due to widespread smartphone possession [5]. Because AACE occurs after the complete development of binocular function, it shows the successful recovery of stereopsis after the surgery [6]. However, studies on the surgical outcomes of only adults are rare. The purpose of this study was to analyze the postoperative prognosis such as angle of deviation and stereoacuity and the factors that affect it, in adult AACE cases.

## Methods

Records of 24 patients diagnosed with AACE who had undergone surgery from October 2012 to March 2020 at the Chosun University Hospital were reviewed retrospectively. AACE of adulthood was diagnosed if esotropia with sudden diplopia developed after 18 years of age and the angle of deviation in near and distance, lateral gaze showed a difference within 5 prism diopters (PD) without limitation of eye movement. Patients with systemic diseases including hyperthyroidism, myasthenia gravis which may cause esotropia, and patients with a past history of strabismus or strabismus surgery were excluded. In addition, patients with hypermetropia were excluded if the esodeviation angle was decreased > 5 PD after hypermetropia correction. This study was conducted in adherence to the tenets of the Declaration of Helsinki and was approved by the Institutional Review board of Chosun University Medical Center.

The following data were abstracted from the medical records: sex, age at onset, age at surgery, manifest refraction and cycloplegic refraction. The refractive values from the cycloplegic refraction were converted to a spherical equivalent (SE) for the statistical analysis. The alternate prism cover test was performed to measure the angle of deviation at near and distance fixation for all gaze directions with and without refractive correction. Ocular movement abnormality and comitancy of esotropia were identified by Lancaster test. Stereoacuity was assessed with the Titmus test card at 40 cm and then converted to arc/sec for statistical analysis. All patients underwent Brain Magnetic Resonance Imaging (MRI) to identify any abnormality in the brain or orbits and general work-ups including blood tests to identify any systemic disease. In addition, patients who used smartphones for more than four hours a day for the last four months have been defined as smartphone users [7].

All of the surgeries were performed under general anesthesia by a single surgeon (DH Kim). The operation chosen either unilateral medial rectus recession and lateral rectus resection (RR) or bilateral medial rectus recession (BMR). The amount of lateral rectus resection and medial rectus recession followed the standard Parks surgical table. Patients were defined as the motor-success group when the deviation angle of strabismus was ≤ 8 PD after the surgery and patients were defined as the sensory-success group when postoperative stereoacuity was 40–60 s. We compared these success groups to the others in terms of age of onset, duration of esotropia, length of preoperative follow-up period, preoperative deviation angle near and distant, preoperative stereoacuity, surgical method, length of postoperative follow-up period, and smartphone use. We analyzed these differences to find what factors affect the surgical outcome. SPSS version 20.0 (IBM corporation, Armonk, NY, USA) was used for the statistical analysis. Fisher’s exact test was used to compare the type of surgery, smartphone use, preoperative stereoacuity and postoperative diplopia. Mann–Whitney U test was used to compare the age at onset, age at surgery, duration of esotropia, preoperative angles of deviation and preoperative and postoperative follow up period. A *p* values of < 0.05 was considered statistically significant.

## Results

The study included 24 AACE of adult patients, 17 were males and 7 were females. The mean age of onset was 25.3 ± 8.6 years (range, 18–49 years). The mean age at diagnosis was 26.3 ± 8.7 years (range, 18–49 years). The mean age of receiving surgery was 26.6 ± 8.7 years (range, 18–50 years). The mean duration from onset to receiving the surgery was 14.3 ± 17.1 months (range, 1–66 months). Each patient presented with a visual acuity of 20/20 in both eyes on their first visit. Most patients had myopia with a mean SE of the refraction of -2.9 ± 2.1 diopter (D) on the right eye and − 3.0 ± 1.8 D on the left eye. One patient was hyperopic (both eyes were + 1.75 D) and another patient was emmetropic. The mean esodeviation angle before surgery was 33.3 ± 11.2 PD (range, 16–60 PD) at near and 33.1 ± 10.4 PD (range, 16–60 PD) at distance. The mean preoperative follow-up period was 3.8 ± 2.7 months (range, 3 weeks–7 months). Among 24 patients, RR was done to 18 patients (75.0%) and BMR was done to 6 patients (25.0%). The mean length of postoperative follow-up was 7.5 ± 4.5 months (range, 1–18 months). On the final follow-up visit, the patients showed mean esodeviation angle of 3.8 ± 6.7 PD (range, − 8 ~ 20 PD) at near and 3.4 ± 6.1 PD (range, − 8 ~ 16 PD) at distance. Before the surgery, 6 patients (25.0%) had no stereopsis, 18 patients (75.0%) had stereopsis. However, all patients recovered their stereopsis after the surgery; 12 patients (50.0%) had 80–400 arc/sec of stereopsis, and 12 patients (50.0%) had 40–60 arc/sec of stereopsis. We did not find any abnormality in the patients’ brain MRI. Out of 24 patients, 13 (54.2%) responded that they used their smartphone for many hours. (Tables 1 and 2).

**Table 1:** Data of individual patients with acute acquired comitant esotropia

Patients No.	Duration of esotropia (month)	Preoperative esodevitaion (PD) (near/distance)	Preoperative steroacuity (sec/arc)	Type of surgery	Presence of diplopia
1	8	40/40	40	RR	Intermittent
2	63	35/35	400	RR	Constant
3	28	18/25	200	RR	Intermittent
4	7	25/25	300	RR	Constant
5	4	20/20	80	RR	Intermittent
6	24	40/40	200	RR	Constant
7	6	25/25	100	RR	Intermittent
8	6	25/30	80	RR	Intermittent
9	8	35/30	200	RR	Constant
10	8	20/25	400	BMR	Constant
11	5	45/40	Nil	RR	Intermittent
12	2	35/35	400	RR	Intermittent
13	19	25/25	200	RR	Constant
14	9	40/40	Nil	RR	Constant
15	66	40/40	Nil	RR	Intermittent
16	2	60/60	Nil	BMR	Constant
17	18	35/35	3000	RR	Intermittent
18	7	40/35	200	RR	Constant
19	7	45/40	Nil	BMR	Constant
20	9	16/14	200	BMR	Intermittent
21	8	20/16	80	BMR	Intermittent
22	4	45/45	Nil	BMR	Constant
23	1	25/30	800	RR	Constant
24	24	45/45	800	RR	Constant

*No* number, *PD* prism diopters, *RR* unilateral medial rectus recession and lateral rectus resection, *BMR* bilateral medial rectus recession

**Table 2:** Characteristics of adult patients who had surgery with acute acquired comitant esotropia

Variables	*N* = 24
Sex (Male : Female)	17 : 7
Age at onset (years)	26.3 ± 8.7
Age at surgery (years)	26.6 ± 8.7
Duration of esotropia (months)	14.3 ± 17.1
Spherical equivalent (Diopter)	
Right	-2.9 ± 2.1
Left	-3.0 ± 1.8
Preoperative angle of esodeviation (PD)	
Near	33.3 ± 11.2
Distance	33.1 ± 10.4
Preoperative stereoacuity	
Stereopsis (-)	6 (25.0%)
Stereopsis (+)	18 (75.0%)
Preoperative follow-up period (months)	3.8 ± 2.7
Type of surgery	
RR	18 (75.0%)
BMR	6 (25.0%)
Postoperative angle of esodeviation (PD)	
Near	3.8 ± 6.7
Distance	3.4 ± 6.1
Postoperative follow-up period (months)	7.5 ± 4.5
Postoperative stereoacuity	
Stereopsis (-)	0 (0.0%)
Stereopsis (+)	24 (100.0%)
Smartphone use	
Yes	13 (54.2%)
No	11 (45.8%)
Preoperative diplopia	
Intermittent	10 (41.7%)
Constant	14 (58.3%)

*PD* prism diopters, *RR* unilateral medial rectus recession and lateral rectus resection, *BMR* bilateral medial rectus recession

Of 24 patients, 19 (79.2%) classified as the motor-success group and the mean esodeviation angle at the final visit was 1.1 ± 3.7 PD at near and 0.9 ± 3.4 PD at distance. 5 patients (20.8%) classified as the motor-failure group and the mean esodeviation angle at the final visit was 14.4 ± 4.3 PD at near and 12.8 ± 4.6 PD at distance. There is no postoperative diplopia except for one of the failure groups. Between the success and failure groups, there was no difference in age at onset, age at surgery, duration of esotropia, length of preoperative follow-up period, preoperative deviation angle at near or distance, preoperative stereoacuity, length of postoperative follow-up period, smartphone use, or postoperative diplopia. However, there was a statistically significantly difference in the surgical method (*p* = 0.006). Of the 18 patients who underwent RR, 17 patients were success group. Meanwhile, of the 5 patients who underwent BMR, 4 patients were failure group (Table 3). The preoperative esodeviation angle and surgical amount were no difference between RR and BMR group (Table 4).

**Table 3:** Comparison of clinical factors between motor success and failure group

Variable	Success Group (*n* = 19)	Failure Group (*n* = 5)	*P*-value
Sex (Male : Female)	16 : 3	1 : 4	
Age at onset (years)	26.1 ± 9.5	22.6 ± 2.8	0.836^b^
Age at surgery (years)	27.5 ± 9.5	23.0 ± 2.9	0.581^b^
Duration of esotropia (months)	16.3 ± 18.8	6.6 ± 2.1	0.629^b^
Preoperative follow-up period (months)	3.7 ± 2.8	4.0 ± 2.1	0.629^b^
Preoperative angle of esodeviation (PD)			
Near	33.1 ± 10.6	34.2 ± 14.9	0.783^b^
Distance	33.7 ± 9.4	31 ± 14.8	1.000^b^
Preoperative stereoacuity			
Stereopsis (-)	3 (15.8%)	3 (60.0%)	0.078 ^a^
Stereopsis (+)	16 (88.9%)	2 (40.0%)	
Type of surgery			
RR	17 (89.5%)	1 (20.0%)	0.006^a^
BMR	2 (10.5%)	4 (80.0%)	
Postoperative follow-up period (months)	8.1 ± 4.9	5.4 ± 1.3	0.367^b^
Smartphone use			
Yes	11 (57.9%)	2 (40.0%)	0.630^a^
No	8 (42.1%)	3 (60.0%)	
Postoperative diplopia			
Yes	0 (0.0%)	1 (20.0%)	0.630^a^
No	19 (100.0%)	4 (80.0%)	

*PD* prism diopters, *RR* unilateral medial rectus recession and lateral rectus resection, *BMR* bilateral medial rectus recession

^a^Fisher’s exact test, ^b^Mann-Whitney U test

**Table 4:** Comparison of the preoperative esodeviation angle and surgical amount for each surgical group

Variable	RR (*n* = 18)	BMR (*n* = 6)	*P*-value
Preoperative angle of esodeviation (PD)			
Near	34.3 ± 10.7	30.2 ± 13.2	0.258^*^
Distance	34.4 ± 9.5	29.2 ± 12.8	0.244^*^
Surgical amount (mm)	10.2 ± 1.4^a^	10.0 ± 1.7^b^	0.899 ^*^

*RR* unilateral medial rectus recession and lateral rectus resection, *BMR* bilateral medial rectus recession, *PD* prism diopters

^a^Mean value of the sum of the amounts for recession and resection

^b^Mean value of the sum of the amounts for medial rectus recession

*Mann-Whitney U test

Of 24 patients, 12 patients (50.0%) classified as the sensory-success group and 12 patients (50.0%) classified as the sensory-failure group. Between the success and failure groups, there was no difference in age of onset, age of surgery, duration of esotropia, length of preoperative follow-up, preoperative deviation angle near and distant, preoperative stereoacuity, or smartphone use. However, the postoperative follow-up period was statistically significantly longer in the success group at 10.3 ± 4.9 months compared to the failure group at 4.8 ± 1.8 months (*p* = 0.006) (Table 5).

**Table 5:** Comparison of clinical factors between sensory success and failure group

Variable	Success Group (*n* = 12)	Failure Group (*n* = 12)	*P*-value
Sex (Male : Female)	9 : 3	8 : 4	
Age at onset (years)	27.1 ± 10.1	25.4 ± 7.4	0.060^b^
Age at surgery (years)	27.4 ± 10.2	25.8 ± 7.3	0.932^b^
Duration of esotropia (months)	14.1 ± 17.3	12.6 ± 17.8	0.843^b^
Preoperative follow-up period (months)	3.9 ± 3.0	3.7 ± 2.4	0.887^b^
Preoperative angle of esodeviation (PD)			
Near	30.3 ± 9.1	36.3 ± 12.7	0.198^b^
Distance	30.8 ± 7.0	35.4 ± 12.9	0.242^b^
Preoperative stereoacuity			
Stereopsis (-)	1 (8.3%)	5 (41.7%)	0.155 ^a^
Stereopsis (+)	11 (91.7%)	7 (58.3%)	
Type of surgery			
RR	10 (83.3%)	8 (66.7%)	0.640^a^
BMR	2 (16.7%)	4 (33.3%)	
Postoperative follow-up period (months)	10.3 ± 4.9	4.8 ± 1.8	0.006^b^
Smartphone use			
Yes	8 (66.7%)	5 (41.7%)	0.219^a^
No	4 (33.3%)	7 (58.3%)	

*PD* prism diopters, *RR* unilateral medial rectus recession and lateral rectus resection, *BMR* bilateral medial rectus recession

^a^Fisher’s exact test, ^b^Mann-Whitney U test

## Discussion

AACE can occur at any age in infancy or adulthood. Nevertheless, the clinical conditions in children and adults are considered different [8]. In adults, AACE usually occurs in myopia patients with normal corrective vision. Several studies reported good surgical outcomes at AACE and binocular vision including stereopsis can be recovered to normal. However, the exact mechanism and clinical features of AACE have not been fully established [8, 9]. In this study, the corrected visual acuity of all patients was normal and most were myopic except two patients. Bielschowsky [10] reported that medial rectus is strengthened in myopia patients resulting in esotropia as convergence amplitudes becomes stronger than divergence amplitudes from near work for a long time without correction by glasses. However, all patients in this study are not likely to be described by the above mechanisms due to performing near work with glasses on. Lee et al. [5] reported that a person with weak convergence amplitudes or esophoria can develop acute esotropia when they have used a video display terminal (VDT) such as a smartphone close up for many hours due to develops abnormalities of accommodations and convergence. However, this does not fully explain the mechanism as only 13 out of 24 patients had used smartphones for many hours in our study. Recently, Ali et al. [9] reported that patients with esophoria have enhanced divergence amplitudes to suppress manifest esotropia and could occur diplopia and manifest esotropia if the divergence amplitude fail to overcome esophoria in adulthood. In our study, we did not measure the divergence amplitudes in patients and did not know whether the above mechanism caused the esotropia. Therefore, further study is needed to determine the mechanism of AACE manifestation.

The surgical outcomes of AACE are not yet established as most previous reports studied a few patients at various ages. In addition, reports of effective surgical methods are rare, so it is not yet well known about which surgery is best. Lyon et al. [11] reported a good surgery outcomes for five AACE patients aged 3–24 years who underwent BMR. Lee et al. [5] reported that three patients aged 7–16 years underwent BMR and showed orthophoria on the final postoperative follow-up. On the other hand, Song et al. [12] found that five out of six AACE patients with age older than 17 years did not have favorable outcomes and still had esotropia of > 18 PD after BMR. Accordingly, Song et al. also suggested the cause of unfavorable outcomes that there were possible unmeasured occult strabismus and structural causes such as medial rectus stricture and lateral rectus fibrosis. In our study also four out of five patients who had undergone BMR were classified as the motor-failure group and did not show favorable surgical outcomes. Because undercorrection was frequent when recession was done by the traditional surgical angle, Ali et al. [9] recommended that surgical target angle be augmented by an additional 10 PD in AACE. Furthermore, Veles and Rosenbaum [13] and Savino et al. [14] showed that the prism adaptation test could find the residual esodeviation angle and the surgical outcome was improved when medial rectus recession was done considering the residual esodeviation angle. In this study, the poor results of BMR may be considered that we did not perform the prism adaptation test and recession was done by the traditional surgical angle. However, in this study 18 patients who underwent RR showed good outcomes except one patient. Kim et al. [15] reported that all 11 patients who underwent RR showed orthophoria on final follow-up while 4 patients who underwent BMR had remaining esotropia of > 10 PD and showed similar results with our study. In addition, in the report of Spierer [8], 4 out of 10 patients with AACE underwent RR, and all 4 patients were had orthophoria, but in 6 patients who underwent BMR, only 2 patients were had orthophoria that RR showed better outcome than BMR. Miles and Burian [16] reproted RR may be more suitable than BMR in respect of surgery for esotropia and suggested that the mechanical muscle reinforcement effect of lateral rectus resection may be more effective in correcting esotropia than the relative strengthening effect of lateral rectus muscle due to the reduction of nerve impulse of medial rectus recession. In this study reported RR showed better outcome than BMR. It can be assumed that the mechanical strenthening effect of lateral rectus resection may be more effective in enhancing the weakened divergence amplitude in AACE patients [17].

AACE occurs after the complete development of binocular function and shows the successful recovery of stereopsis after correction esotropia by surgery. However, reports on the postoperative stereopsis of AACE cases in adulthood are relatively rare and the most reports until now have been based on cases of acquired esotropia in childhood. Ohtsuki et al. [18] reported that 15 (60%) of 25 acquired esotropia patients at mean age 12 years recovered stereopsis of more than 60 seconds and the duration of esotropia or age of onset did not significantly affect the recovery. In contrast, Kassem and Elhilali [19] reported that the possibility of binocular vision recovery was higher when the duration of esotropia was shorter and the age of surgery was younger in acquired esotropia cases of mean age 8.6 years. In particular, the duration of esotropia is the most influential factor on the stereopsis recovery in esotropia patients including accommodative esotropia [20]. In our study, which is based on adult AACE patients, there was no significant difference in esotropia duration between the patient group with recovered stereopsis ≥ 60 arc/sec and the group with stereopsis < 80 arc/sec. However, the group with recovered stereopsis had a longer postoperative follow-up period. This result accords with Sturm et al. [21], which was based on pediatric patients. They reported that it may take around 18 months for pediatric patients to recover normal stereopsis after esotropia correction surgery. Therefore, 12 patients who did not recover stereopsis of ≥ 60 arc/sec in this study may need further follow-up to identify improvements of stereopsis. Additionally, some patients in this study may have decompensated monofixation syndrome among those who failed to recover within 60 arc/sec, as Savino et al. [14] reported that the stereopsis of monofixation syndrome did not recover after the surgery compared to AACE. However, Ali et al. [9] asserted that all AACE patients with decompensated esophoria could recover normal stereopsis of ≥ 60 arc/sec. In this study, If patients did not recover normal stereopsis in additional follow-up, we must consider the possibility of decompensated monofixation syndrome.

This study had some limitations. First, we cannot presented a control group consisting of adult esotropia with other causes to compare the use of RR and BMR, so it is difficult to generalize whether the results are limited to AACE or not. Second, the sample size was not big enough and the number of patients differed significantly between RR and BMR group, so need to be very careful to interpret the results. Third, because all patients had diplopia before surgery, the preoperative stereoscopic value was not accurate. Additionally follow-up period was not long enough to detect that patients who failed to achieve stereoacuity could eventually achieve stereoacuity. Moreover, there was a large difference in the number of patients between the motor-success and failure groups, the criteria of selecting the surgical method was not randomized, the hours of smartphone use were imprecise as they depended on patients’ memories and subjects may have included decompensated monofixation syndrome patients since the age of onset also depended on patients’ memory. To overcome these limitations, further studies with a larger number of patients and long-term follow up should be performed.

## Conclusions

The surgical outcomes of adult AACE would be successful when the suitable surgery method is applied. Furthermore, postoperative stereopsis is expected to recover favorably but full recovery may take longer; therefore, we recommend long-term postoperative follow-up.

## Data Availability

The data are available from the corresponding author upon reasonable request.

## Abbreviations

AACE: Acute acquired comitant esotropia
PD: Prism diopters
SE: Spherical equivalent
MRI: Magnetic Resonance Imaging
RR: Unilateral medial rectus recession and lateral rectus resection
BMR: Bilateral medial rectus recession
